# The Dynamic Impact Response of 3D-Printed Polymeric Sandwich Structures with Lattice Cores: Numerical and Experimental Investigation

**DOI:** 10.3390/polym13224032

**Published:** 2021-11-21

**Authors:** Shu-Yu Jhou, Ching-Chi Hsu, Jui-Chia Yeh

**Affiliations:** 1 Graduate Institute of Applied Science and Technology, National Taiwan University of Science and Technology, Taipei 106, Taiwan; yu78617@gmail.com; 2 Department of Mechanical Engineering, National Taiwan University of Science and Technology, Taipei 106, Taiwan; 3 Footwear & Recreation Technology Research Institute, Taichung 407, Taiwan; 0811@bestmotion.com

**Keywords:** sandwich structure, lattice, finite element, dynamic impact, shock absorption, collapse

## Abstract

This paper proposes a dynamic drop weight impact simulation to predict the impact response of 3D printed polymeric sandwich structures using an explicit finite element (FE) approach. The lattice cores of sandwich structures were based on two unit cells, a body-centred cubic (BCC) and an edge-centred cubic (ECC). The deformation and the peak acceleration, referred to as the g-max score, were calculated to quantify their shock absorption characteristic. For the FE results verification, a falling mass impact test was conducted. The FE results were in good agreement with experimental measurements. The results suggested that the strut diameter, strut length, number and orientation, and the apparent material stiffness of the lattice cores had a significant effect on their deformation behavior and shock absorption capability. In addition, the BCC lattice core with a thinner strut diameter and low structural height might lead to poor shock absorption capability caused by structure collapse and border effect, which could be improved by increasing its apparent material stiffness. This dynamic drop impact simulation process could be applied across numerous industries such as footwear, sporting goods, personal protective equipment, packaging, or biomechanical implants.

## 1. Introduction

A sandwich panel is a structure consisting of a thin skin-layer bonded to each side of a lower density core. With widely used additive manufacturing, the core structure of the sandwich panel is possible to fabricate as a complex cellular material in stainless steel and metal alloys [1,2,3], as well as in elastomer and polymers [4,5], which cannot be manufactured by traditional manufacturing technologies. Cellular material has shown its potential for the applications of impact energy absorption [6,7,8,9,10], such as packaging and protective devices [3,11,12,13], while maintaining relatively high stiffness and low density. Moreover, there is a growing interest in 3D printed polymeric cellular material that attempts to improve shoe designs and performance characteristics, such as cushioning, in the footwear industry [14,15,16]. A lattice structure is one type of cellular materials constructed by uniform and ordered unit cells. The mechanical properties of the lattice structures are determined by their architecture, such as cell type, cell size, and strut diameter [11,17,18,19], rather than material composition.

Most experimental and numerical studies had investigated the mechanical behavior and energy absorption capability of the 3D printed polymeric lattice structure under quasi-static compression or dynamic compression. Habib et al. (2018) [7], Abate et al. (2020) [20], Alwattar et al. (2019) [21], Chen et al. (2020) [22], and Dar U.A. et al. (2020) [23] performed an experimental and computational investigation of lattice structure with a variety of unit cell designs under quasi-static compression loading. In their computational simulation, the LS-DYNA implicit solver, ABAQUS, and ABAQUS explicit package were, respectively, used to predict the compressive behavior or stress-strain curves. Bai L. et al. (2020) [24] performed a dynamic compression test and simulation with different loading speeds to investigate the effect of lattice structure densities and configurations on its compression responses. In addition to the quasi-static compression and dynamic compression testing, Ling et al. (2019) [25], Davami et al. (2019) [26], and Rifaie M.A. et al. (2019) [27] conducted a low-velocity impact test, which allowed the impactor to drop from a specific height to observe the dynamic impact response of the lattice structures with different cell designs and materials. The force-time history curve, energy, and deformation of the lattice structures were calculated to quantify their shock absorption capability. Although some experimental studies have performed low-velocity impact test to study the shock absorption capability of 3D printed polymeric lattice structures, few numerical studies have examined their dynamic impact response.

In the present study, a dynamic drop weight impact simulation using an explicit FE approach was proposed to predict the impact response of the sandwich structures with various lattice cores. Particular attention was given to evaluate how the unit cell design, strut diameter, and apparent material stiffness of the lattice core affected its energy absorption capability and structural collapse. The thermoplastic polyurethane (TPU 95A) sandwich structures with lattice core were 3D printed and modelled. ANSYS LS-DYNA with explicit solver was used to predict their impact response. Falling mass impact standard tests were performed to validate the FE results. The deformation and peak acceleration, referred to as the g-max score, were calculated to quantify the shock absorption characteristic of sandwich structures under a dynamic impact loading. The FE results were compared with the experimental measurements.

## 2. Materials and Methods

### 2.1. Design of Lattice Structure

**Figure 1:**
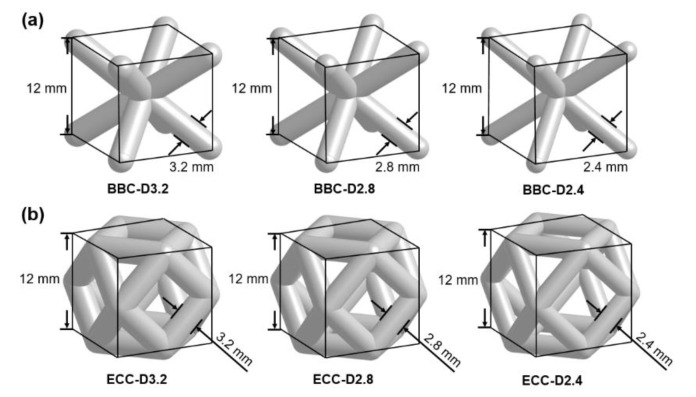
Geometry definition for two individual strut-based unit cells: (**a**) body-centered cubic (BCC) unit cell, and (**b**) edge-centered cubic (ECC) unit cell.

**Figure 2:**
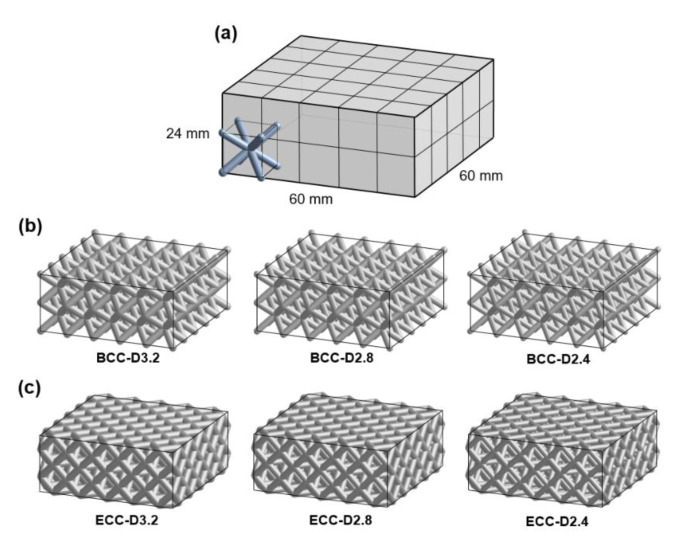
(**a**) Definition for the block of lattice core sample, and the lattice core samples constructed from (**b**) BCC unit cells and (**c**) ECC unit cells.

This study focuses on sandwich panels, a thin skin-layer bonded to each side of lattice core structures consisting of linearly repeating unit cells in all directions. Two cubic strut-based unit cells, body-centered cubic (BCC) unit cell and edge-centered cubic (ECC) unit cell named after crystalline structures, were selected to construct the lattice cores. The configuration and porosity of the lattice core were changed by altering the strut diameter while maintaining the same cell size. The individual cell size was 12 mm, and strut diameters were 3.2 mm, 2.8 mm, and 2.4 mm (Figure 1). Each lattice core consisted of 50 cells, 5 × 5 × 2 in three directions to obtain a building block with dimensions 60 × 60 × 24 mm in Figure 2a. Figure 2b,c show the image of six lattice cores developed using the software ANSYS DesignModeler 19.2 (ANSYS, Inc., Canonsburg, PA, USA) in this study for investigating the differences in their dynamic impact response. The porosity is also a key feature to describe the characterization of the lattice core [2], and an important parameter for lattice structure topology optimization. It is defined as the ratio of the pore volume to that of its solid sample. Table 1 lists the porosity of the six cubic based lattice cores. 

### 2.2. Explicit Finite Element Modelling

**Figure 3:**
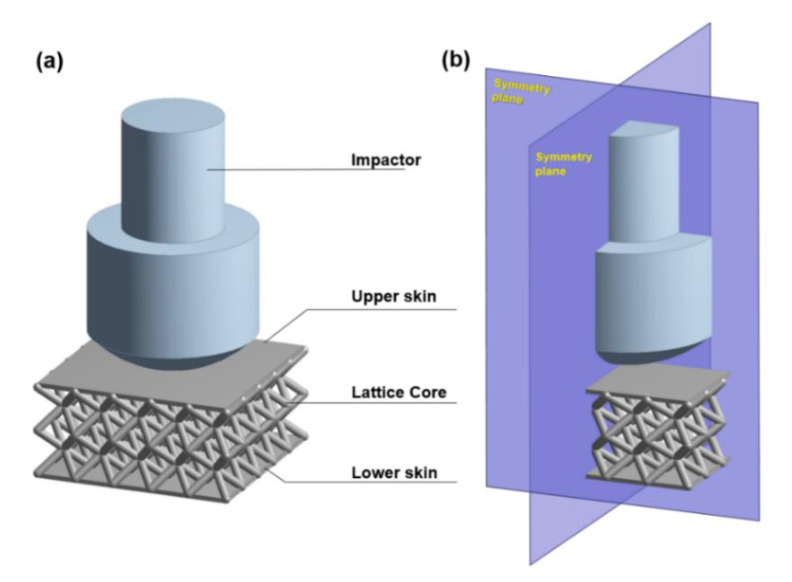
(**a**) The structure of 3D finite element model, and (**b**) a quarter-symmetry model for this analysis.

To better understand the cushioning mechanism of the sandwich panel with six types of lattice core, ANSYS LD-DYNA with the explicit solver was employed to simulate their dynamic impact response following SATRA TM142—Falling mass shock absorption test [28]. A 3D FE model consists of an upper skin, lower skin, lattice core, and impactor (Figure 3a). A quarter-symmetry model shown in Figure 3b was used for this analysis. The dimension of the upper and lower skins was 60 × 60 × 1.2 mm, and the specification of the impactor was based on the SATRA STM479 dynamic shock absorption test machine developed to conduct the SATRA TM142 test method. The design of the lattice structure described in Section 2.1 was used as the geometric property of the lattice core model. The tetrahedron elements were used to describe the entire model rather than modelling the skins with shell elements or the lattice cores with beam elements. An optimal mesh density determined based on convergence analysis was used to simulate models in a reasonable time. TPU 95A, a common 3D printing material with high flexibility, elasticity, and shock resistance [29], was applied for the skins and lattice core. The uniaxial compression test was conducted to define the material behavior of TPU 95A [30]. The material property of the rigid impactor was based on the specification of the SATRA STM479 dynamic shock absorption test machine. The physical properties of the sandwich structures and impactor are presented in Table 2. To further explore the effect of apparent material stiffness on energy absorption capability, Young’s modulus of the sandwich structure was adjusted at 18, 54, 72, and 144 MPa.

### 2.3. Dynamic Simulation and Data Output

**Figure 4:**
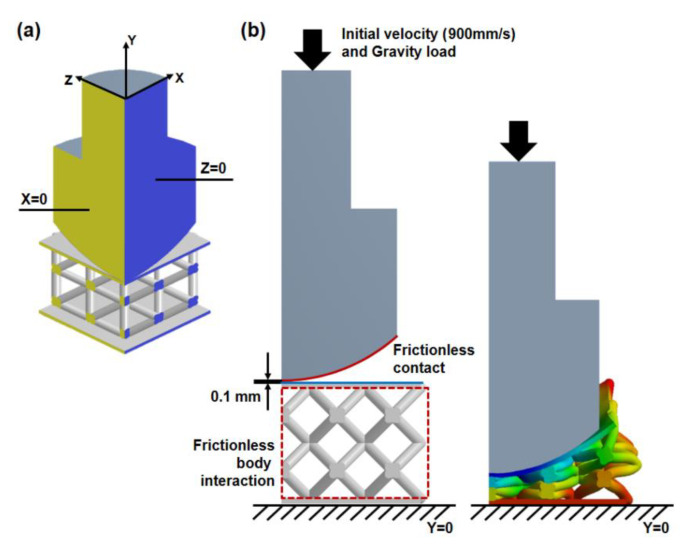
(**a**) Symmetry boundary conditions, and (**b**) loading and contact conditions.

**Figure 5:**
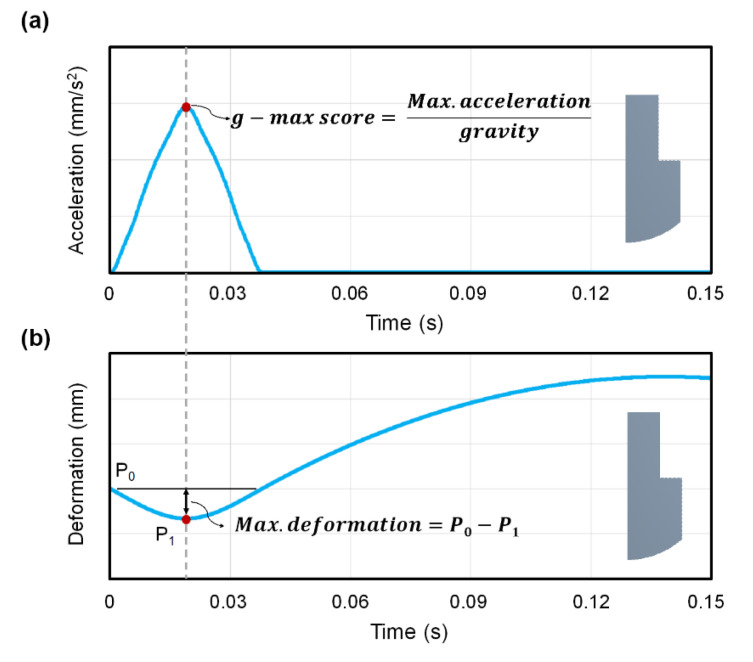
The definition of data output (**a**) g-max score, and (**b**) maximum deformation.

The simulation was performed by allowing the impactor to fall onto the sandwich structure from a defined height. With the quarter-symmetry FE model, the definition of symmetry regions was required. Displacement is fixed in directions perpendicular to the plane of the cut as illustrated in Figure 4a. The vertical displacement at the bottom surface of the sandwich structure was fixed when the impactor was allowed to fall and rebound only in the vertical direction. Compared to the mechanical test, the defined height of the impactor above the sandwich structure was moved down from 50 mm to 0.1 mm in simulation to minimize the time required for calculation. An initial velocity of 990 mm/s and gravity load was applied to the impactor to mimic the SATRA TM142 shock absorption test. Interaction of the sandwich structure with itself and frictionless contact between the impactor and top plate were applied to prevent the penetration of contacting surfaces during the simulation (Figure 4b). In this case, the end time of this simulation process was set as 0.15 s. In post-processing, the acceleration of the impactor and the deformation of the sandwich structure in vertical direction were calculated for 1500 equally space point during the problem-solving cycle to determine the shock absorption capability as shown in Figure 5. The g-max score was defined as the ratio of the peak acceleration of impactor to acceleration due to gravity [31]. A lower g-max score indicates better shock absorption ability of the structure.

### 2.4. Mechanical Shock Absorption Test

**Figure 6:**
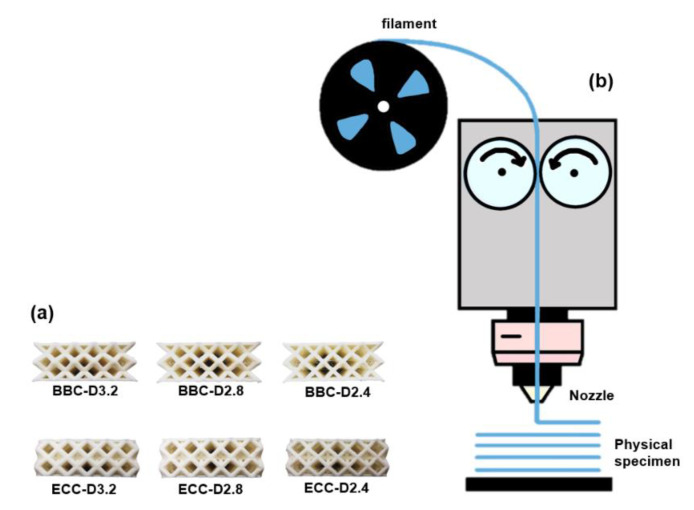
(**a**) The TPU 95A specimens of sandwich structures with six types of lattice core fabricated by (**b**) FDM manufacturing technique.

For the FE results verification, the six specimens in Figure 6a were fabricated using fused deposition modelling (FDM), one of the most widely used additive manufacturing techniques, with a flexible polymer TPU 95A (Footwear & Recreation Technology Research Institute, Taichung, Taiwan) for the experimental process. The main benefits of 3D printing enable the operator to make previously impossible geometries and improve the efficiency of the development process. Especially for the FDM technique, the prototypes can be produced most affordably. Creality CR-10 V2 3D printer (Creality, Co., Ltd., Shenzhen, China) was used to operate the FDM process. During the FDM process, each specimen was built by heating a TPU 95A filament with a diameter of 1.75 mm to 190 °C and extruding from 0.6 mm nozzle size layer by layer with the printed speed of 20 mm/s until the specimen was formed (Figure 6b).

**Figure 7:**
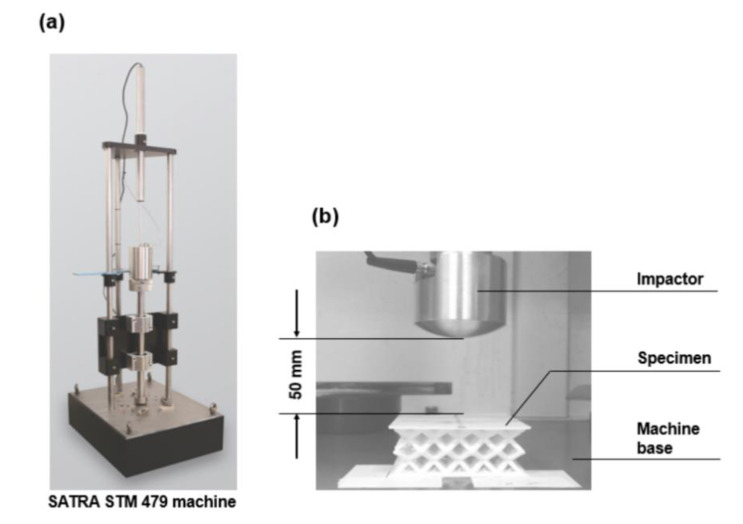
(**a**) The SATRA STM 479 machine for the falling mass shock absorption tests, and (**b**) the experimental setup.

The falling mass shock absorption tests of the sandwich panel, according to SATRA TM142, were conducted using the SATRA STM 479 machine to validate the feasibility of numerical analysis (Figure 7a). Before performing any tests, a horizontal surface was needed on which to place the testing machine. All the specimens were held in position on the machine base by affixing double-sided tape to their bottom surfaces, and an 8.5 kg mass of impactor was allowed to fall freely at a drop height of 50 mm (Figure 7b). The displacement of the specimen and the g-max score for the impactor were determined by the accelerometer and displacement transducer incorporated into the machine. Each test was repeated five times for the average maximum deformation for the specimen and the g-max score for the impactor.

## 3. Results

**Figure 8:**
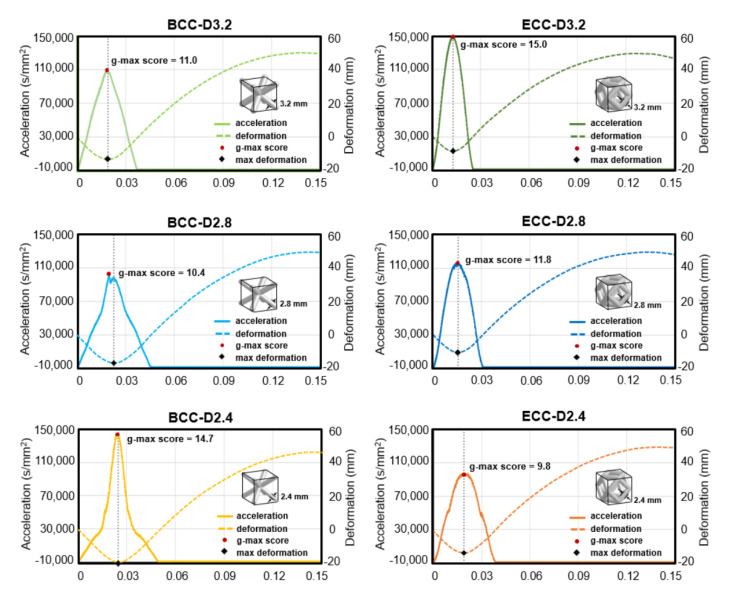
The acceleration-time and deformation-time curves of the sandwich structures with six types of lattice core under dynamic impact.

The dynamic impact displacement-time and acceleration-time diagrams of six sandwich structures with original TPU 95A material obtained from the FE method are presented in Figure 8. Deformation behavior, max deformation, acceleration response, and g-max score are shown in each diagram and will be discussed in the following sections.

### 3.1. Deformation Response

**Figure 9:**
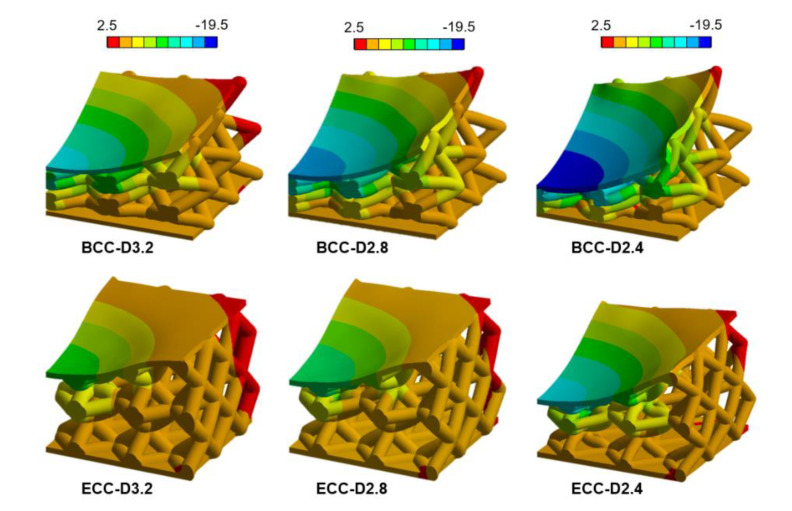
The maximum deformation of the sandwich structures with six types of lattice core under dynamic impact.

From the displacement-time curves (dotted line) in Figure 8, it can be seen that the maximum deformation of the sandwich structures with lattice cores is relevant to their strut diameter and cell shape. For both BCC and ECC unit cells, the maximum deformation of the sandwich structures with lattice cores increased by reducing their strut diameter. The strut diameter of 2.4 mm showed the highest maximum deformation of BCC and ECC unit cells at 19.6 mm and 13.5 mm, respectively. The strut diameter of 3.2 mm showed the lowest maximum deformation of BCC and ECC unit cells at 13.2 mm and 9.0 mm, respectively. Based on the same strut diameter, the maximum deformation of the BCC unit cell was greater than that of the ECC unit cell. From the impact deformation distributions of sandwich structures with six types of lattice core in Figure 9, it was found that a significant structural collapse occurred in BCC-D2.8 and BCC-D2.4.

### 3.2. Shock Absorption Ability

To quantify the magnitude of the shock-absorbing capability of sandwich structures, the peak acceleration, which is commonly referred to as the g-max score, was determined from the acceleration-time curves (solid line) in Figure 8. A lower g-max score indicates better shock absorption ability of the structure. For both BCC and ECC unit cells, the g-max score of the sandwich structures with lattice cores decreased with decreasing strut diameter, except the BCC unit cell with strut diameter of 2.4 mm. The ECC unit cell with strut diameter of 3.2 mm was 15, the highest g-max score, whereas the ECC unit cell with strut diameter of 2.4 mm showed 9.8, the lowest g-max score. In contrast, the BCC unit cell with strut diameter of 2.8 mm had a slightly lower g-max score than that of the strut diameter of 3.2 mm, but the BCC unit cell with strut diameter of 2.4 mm showed the highest g-max score among BCC unit cells.

### 3.3. Experimental Validation

**Figure 10:**
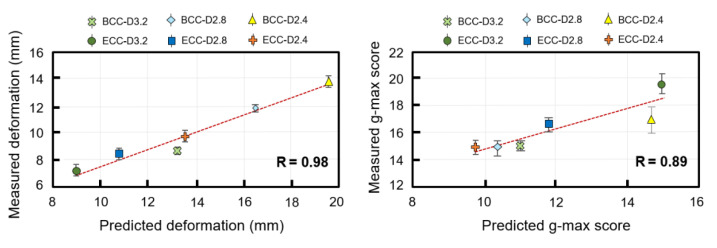
The relationship between experimental measurements and simulation predictions.

The predicted results from the FEA method in this study were in good agreement with measurements from the six types of sandwich structure with lattice core. Figure 10 shows that strong positive correlations existed between numerical predictions and experimental measurements both in deformation and g-max score. The correlation coefficient (R) of the deformation was 0.98, whereas the correlation coefficient of the g-max score was 0.89. However, the FEA method predicted a higher deformation and lower g-max score than the experimental measurements.

### 3.4. Apparent Material Stiffness Effect

**Figure 11:**
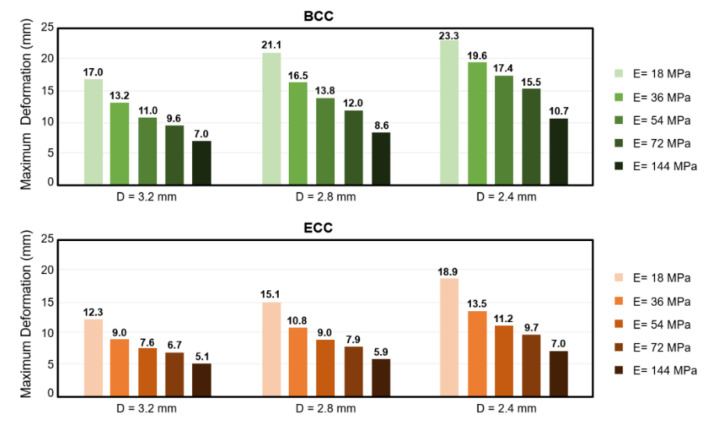
The comparison of the maximum deformation of sandwich structures with different material properties.

**Figure 12:**
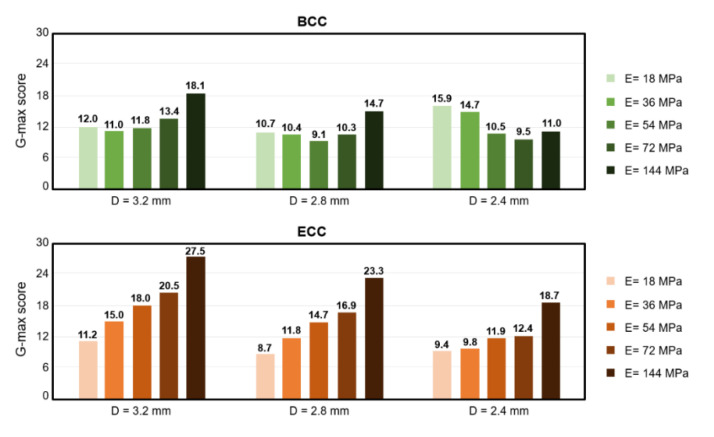
The comparison of the g-max score of sandwich structures with different material properties.

In this section, the effect of apparent material stiffness on the absorption ability of the sandwich structure with lattice core was examined. Figure 11 and Figure 12, respectively, compare the maximum deformation and g-max value predictions of sandwich structures under different material properties adjusted by Young’s modulus (E). In Figure 10, the results show that for both BCC and ECC unit cells, the maximum deformation of the sandwich structure with lattice core decreased by increasing its Young’s modulus, and strut diameters of 3.2, 2.8, and 2.4 mm experienced the same trend. However, different trends were observed in the g-max score of BCC and ECC unit cells (Figure 12). Although the maximum deformation decreased by increasing Young’s modulus, the opposite was true in the g-max of ECC unit cell. The g-max of the sandwich structure with ECC lattice core increased by increasing its Young’s modulus and the same trends existed in strut diameters of 3.2 mm, 2.8 mm, and 2.4 mm. The minimum values of g-max score in ECC unit cells with strut diameter of 3.2 mm, 2.8 mm, and 2.4 mm existed for a Young’s modulus of 18 MPa. For BCC unit cells with strut diameters of 3.2 mm, 2.8 mm, and 2.4 mm, the g-max score decreased to reach a minimum value, and subsequently increased with increasing Young’s modulus. The minimum values of g-max score in BCC unit cells with strut diameters of 3.2 mm, 2.8 mm, and 2.4 mm existed for a Young’s modulus of 36 MPa, 45 MPa, and 74 MPa, respectively.

## 4. Discussion

This study conducted the first numerical simulation to investigate the absorption behavior of sandwich structures with several lattice cores under dynamic impact loading. Due to the limitation of numerical software, the previous studies [7,8,20] tended to predict absorption behavior under quasi-static compression loading. The cumulative area under each compressive stress-strain curve was calculated to evaluate the energy absorption capability. Although this method can be used to compute in a reasonable time and avoid numerical instabilities, the inertial load was neglected. To mimic the impact conditions, the present study considered the inertial load and determined the g-max score at the peak acceleration to evaluate the energy absorption capability.

The cell design, including strut number and orientation, strut diameter, and apparent material stiffness of the lattice cores, were considered as contributing factors in its absorption behavior. In terms of deformation behaviors, our numerical predictions showed that the ECC unit cell provided less deformation than the BCC unit cell due to its strong structure and short strut. The comparison of the results for thin and thick strut structures suggested that decreasing the strut diameter resulted in more deformation. For the energy absorption capability, the ECC unit cell, which was not easily deformed, provided a higher g-max score than the BCC unit cell, which resulted in a poor energy absorption capability. The same results were also reported by previous studies [8,25]. However, the trend noted above did not exist in all conditions. It was observed that the BCC lattice core with thinner strut diameter had poor absorption capability and a higher g-max score. This is due to the structure collapse caused by the significant softening of the BCC unit cell. To further study how the strength of structure and structure collapse affect energy absorption capability, Young’s modulus of the sandwich structure was adjusted at 18, 54, 72, and 144 MPa. In terms of deformation behavior, the ECC unit cell provided less deformation than the BCC unit cell, and the thinner strut diameter resulted in more deformation among five types of structural strength. It was observed that for higher structure strength and a higher value of Young’s modulus, there was less deformation. For the energy absorption capability, the ECC lattice core with thinner strut diameter or lower structure strength had a lower g-max score, which resulted in better absorption capability. As noted above, the BCC lattice core with thinner strut diameter had poor absorption capability and a higher g-max score due to its structure softening. It was observed that the BCC lattice core with thinner strut diameter had a lower g-max score, when the Young’s modulus was 72 and 144 MPa. Our research has revealed that the structure collapse of the BCC lattice core with thinner strut diameter could be improved by increasing the Young’s modulus of its apparent material. In terms of the FE models, the beam element models are computationally inexpensive and used to simulate cell structures in a reasonable time, whereas the solid models allow for a more accurate description of structures’ mechanical behavior [32,33]. Previous studies comparing the beam element FE models and the solid FE models with experiments have shown that the solid FE models provided better prediction than that of the beam element FE models [25,32,34]. Hence, the three-dimensional solid models were used instead of beam element models in this study. The predicted results in this finite element study were in good agreement with experimental results measured from the six types of sandwich structure with lattice core. However, the predicted results revealed a lower g-max score and more structure deformation, which might overestimate the absorption performance. The difference between experimental results and finite element results might be caused by the material property. It is difficult to accurately identify the material property of TPU due to the strain rate effect. In this study, the compression tests were carried out under a displacement control with constant velocity to define the material property of TPU 95A, which was applied to the impact finite element analysis. Unlike the compression tests with constant velocity, the strain rate is not constant under the dynamic impact. Previous studies proved that the strain rate had a significant influence on the material behavior, and a higher strain rate could lead to a stiffer response of the structure [35,36,37]. However, the strain rate effect was not considered in this dynamic impact finite element analysis. In addition to the apparent material property of lattice structure, the quality of the connection between adjacent unit cells contributed to the difference between experimental results and finite element results. The previous study proved that the contact formation between layers of lattice structure has a potential impact on the mechanical performance of the 3D-printed object because of the polymer chain interdiffusion [38]. However, the interface between adjacent unit cells was considered as perfect in this dynamic impact finite element analysis. As a result, the predicted results in this study revealed the softer response of the sandwich structure.

Certain limitations of this research should be noted. To begin with, a linear isotropic elastic material model was assigned to the sandwich structures with various lattice cores. Thus, the deformation and peak g-max score of the sandwich structures might be overestimated under the impact loading. The use of non-linear hyperelastic material models would improve the numerical prediction. The other potential limitation lies in the perfection of the FE model and simulation environment. Ideal or perfect 3D printed sandwich structures were assumed. Thus, surface roughness and dimensional error of the physical prototypes were not considered. Instead of applying double-sided adhesive tape on the contact surface between the sandwich structure and machine base, the FE models were perfectly constrained on the bottom surface and symmetric plane of the sandwich structure. Air resistance was also not considered during the free fall of the impactor.

**Figure 13:**
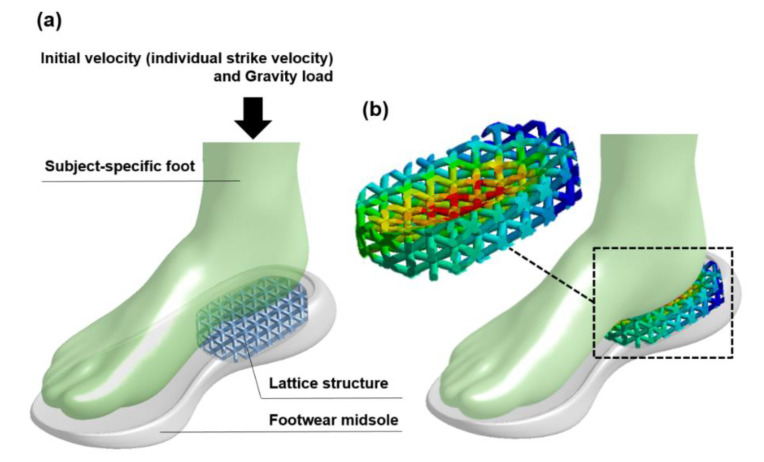
The application of the dynamic impact simulation process on the foot-footwear impact analysis. (**a**) The structure and loading conditions, and the (**b**) lattice structure deformation of the foot-footwear impact analysis.

This study demonstrated the dynamic impact simulation process with an explicit solver to analyze the shock absorption of the 3D printed polymeric sandwich structures. In addition to the simulation technique, the potential application should be further emphasized. As footwear companies has been moving toward custom footwear, 3D printed lattice structures are increasingly adopted in footwear midsoles or other shoe components. In the fabrication process, a previous study proposed a heuristic procedure to determine the optimal values of key process parameters by using a wider range of engineering-grade materials [39]. This optimization method could be integrated with our dynamic computational process to investigate the shock absorption capability of the midsole comprising the lattice structures by considering the various key process parameters, subject-specific foot model, and kinematic and kinetic data, as shown in Figure 13. The benefits of this dynamic impact analysis are clearly illustrated by increasing the shock absorption performance of the lattice structure midsole for an individual foot to support the footwear industry.

## 5. Conclusions

This study aimed to predict the impact response and quantify the shock absorption capability of 3D printed polymeric sandwich structures under a dynamic impact loading. The deformation and the g-max score of the sandwich structures with six types of lattice cores were evaluated and compared. The results of this study revealed the effects of cell design (strut number, length, orientation), strut diameter, and apparent material stiffness of the lattice core on their absorption behavior. The findings suggested that with the stiffer cell shape design, ECC, a thinner strut diameter structure showed better absorption capability. In contrast, with the softer cell shape design, BCC, a thinner strut diameter structure had poor absorption capability due to the structure collapse effect. The collapse of structure can be improved by increasing the Young’s modulus of its apparent material. This dynamic impact study provides understanding of 3D printed polymeric sandwich structures, which can be applied across a large number of industries such as footwear, sporting goods, personal protective equipment, packaging, or biomechanical implants.

## Figures and Tables

**Table 1:** The porosity of the six cubic based lattice structures.

Cell Type	Sturt Diameter (mm)	Porosity (%)
BCC	3.2	72.7
BCC	2.8	78.3
BCC	2.4	83.5
ECC	3.2	65.4
ECC	2.8	72.1
ECC	2.4	78.5

**Table 2:** The physical properties of the sandwich structures and impactor.

	Density (kg/m^3^)	Young’s Modulus (MPa)	Poisson’s Ratio
Skin	1145	36	0.3
Lattice core	1145	36	0.3
Impactor	98,934	200,000	0.3

## Data Availability

The data presented in this study are available on request from the corresponding author.
